# Bridging early medical education and health systems improvement: a multi-faceted faculty development program to enhance engagement and impact

**DOI:** 10.1186/s12909-025-07579-9

**Published:** 2025-07-25

**Authors:** Catherine Y Lau, Edgar Pierluissi, Kristin Casey Callaghan, Anna Chang, Lei Choi

**Affiliations:** 1 Department of Medicine, University of California San Francisco, 521 Parnassus Avenue, #5602, CA San Francisco, 94117 USA; 2 University of California San Francisco, San Francisco, USA; 3 School of Medicine, University of California San Francisco, San Francisco, USA

**Keywords:** Health systems science, Quality improvement, Faculty development, Undergraduate medical education, Experiential learning, Workplace learning

## Abstract

**Background:**

Despite calls to further incorporate health systems science in undergraduate medical education, the ability for early medical learners to participate in authentic, project-based learning in health systems science and improvement has been limited by the knowledge, skills, and competency of teaching faculty. This study explores the impact and outcomes of a comprehensive, longitudinal faculty development program for physician educators in health systems improvement (HSI).

**Methods:**

The University of California, San Francisco HSI faculty development program began with a medical school curricular redesign to include a 14-month weekly course that integrates HSI experiential learning with clinical skills training for early medical students in small groups led by physician coaches. Most physician educators began the role with no formal training in HSI – a gap this program addresses through a multi-faceted approach with standardized and individualized components. We studied the efficacy of the HSI faculty development program and report outcomes using the Kirkpatrick Model, focusing on impact on physicians, students, and health systems.

**Results:**

From 2016 to 2023, the HSI faculty development program reached 119 faculty across 15 departments and three health systems. For Kirkpatrick level one (satisfaction), faculty participated in at least three workshops per year, with an average rating of 4.7 (scale 1–5). Faculty satisfaction in the teaching role is 4.5 and student rating of faculty is 4.8. Each year, faculty demonstrated Kirkpatrick level two (knowledge) by designing up to 60 HSI projects for the class of incoming first year medical students (*n* = 165). Faculty assessed other students’ projects with high concordance with curricular leader grading (> 90%). All students met expectations by course conclusion. For level three (behavior), faculty independently led 35 three-hour small group sessions yearly for a total of 1,281 early medical students over 8 years, and all student project teams completed at least one full Plan-Do-Study-Act cycle. For level four (impact), faculty and students have completed 242 HSI projects to date, each aligned with health system priority areas.

**Conclusions:**

Our study demonstrates that a longitudinal, multi-component HSI faculty development program provides physician faculty sufficient competence to effectively engage medical student-led teams in systems improvement in the clinical learning environment.

**Supplementary Information:**

The online version contains supplementary material available at 10.1186/s12909-025-07579-9.

## Background

The fundamental approach and guiding principles of undergraduate medical education have shifted rapidly over the past two decades, in part driven by the alarming realization of patient safety, quality, health and healthcare equity gaps exposed in the Institute of Medicine’s landmark reports *To Err is Human*, *Crossing the Quality Chasm,* and *Unequal Treatment *[1–8]. Medical school curricular redesign efforts are based on the belief that preparing future physicians to capably navigate and effectively improve health systems to benefit patients and communities requires expansion beyond traditional disciplines to incorporate health systems science, which includes quality improvement, value-based care, and interprofessional teaming [9–12]. Effective redesign will also need to add hands-on “supported participation” in the clinical workplace, beyond lecture halls [13–15]. Thus, physicians-in-training must rely on educators for teaching and coaching that is needed to promote a lifelong habit of applying principles of health systems science towards health systems improvement (HSI) of quality, patient safety, value, and patient and provider experience in their daily practice to address the expanding, complex, and challenging problems facing American health care.

A faculty development program for HSI should be designed with best-practices for competency-based medical education in mind. For instance, faculty development embedded as workplace learning as opposed to separate workshops and didactics can increase faculty participation, motivation, and access to participate in longitudinal communities of learning and practice [16–18]. While communities of practice are effective to promote learning, they are also useful modalities to ensure institutional support, promote sustainability, and support the evolution of professional identities amongst faculty participants [19]. The visibility and value of educators is enhanced when faculty development focuses on organizational development and change that enables participants to effectively contribute to important organizational strategic areas [20]. Thus, a faculty development program centered around an experiential longitudinal HSI curriculum aligns with health system leaders’ values and can catalyze organizational change [20, 21].

However, programs offering faculty the knowledge, tools, and competencies in health systems science often do not adhere to the best-practices of competency-based faculty development and/or are insufficient in depth or practical application within health systems operations, therefore limiting progress toward achieving the aim of effectively engaging early medical learners in HSI [22–25]. Some HSI programs engaging medical learners focused on educator outcomes upstream from impact on learners, and others, vice versa [25–28]. Others focused on later learners, virtual learning, or simulation, and did not have faculty development reported or described [29–31]. Previously published health systems science faculty development programs show improved self-perceived value and/or resulted in the completion of a faculty-led HSI project, but were not embedded within the required student curriculum or health system [19, 20]. It is not known whether participants went on to teach and mentor undergraduate learners in health systems science or on quality improvement teams addressing quality gaps in their local health care systems. Furthermore, the impact of the longitudinal partnership between medical education leadership, physician faculty, and health system leadership was not reported [26, 28, 32].

We conducted this exploratory study to address the following question and knowledge gap: What can be achieved with an evidence-based and multi-component physician faculty development program created in partnership between the medical school and health systems, with the aim of embedding all early medical students in clinical learning environments for HSI? This study frames the findings using all levels of the Kirkpatrick model, with a focus on level four: impact outcomes on the educators, the learners, and the health systems.

## Methods

### Aim and design

This is a prospective, observational, multi-component, program evaluation study to explore the impact of a longitudinal physician educator faculty development program on health systems improvement.

## Setting and participants

At the University of California San Francisco (UCSF) School of Medicine, a required HSI curriculum for all first-year (MS1) and second-year (MS2) medical students was launched in 2016 as a part of the Clinical Microsystems Clerkship (CMC), a longitudinal curriculum that integrates HSI and interprofessional collaboration into the patient care clinical skills curriculum [33]. Approximately 165 MS1s (plus 165 MS2s) are simultaneously embedded in workplace learning communities consisting of one physician faculty coach and 5–6 medical students at three major affiliated health systems (academic, public safety net, and veterans’ affairs). There are approximately 60 concurrent physician faculty coaches funded for their role (20% FTE), which includes delivering and teaching the HSI curriculum [34]. Physician coaches are chosen for their diverse clinical and educational backgrounds and strengths primarily as coaches and mentors. Most coaches do not enter the program with prior training or expertise in health systems improvement.

The HSI curriculum occurs 3 h weekly for a total of 35 sessions spanning 14 months from August of the MS1 year to September of the MS2 year. The vast majority of the curriculum is spent in experiential learning that is primarily designed and guided by coaches, with students learning and applying principles of systems science, including structured problem-solving and change management, while contributing to interdisciplinary teams engaged in improving care. Faculty coaches are responsible for guiding and supporting medical students through the entire systems improvement project on a weekly basis, ensuring adherence to curricular timelines and learning objectives. Curricular elements that are designed by curriculum leaders include introductory lectures and accompanying small group workshops in quality improvement, health systems improvement and A3 problem solving thinking, human centered design, and health and healthcare equity. A health systems improvement symposium occurs at the end of the curriculum to celebrate and showcase the achievements of the faculty-coach led student teams.

## Program components

**Fig. 1:**
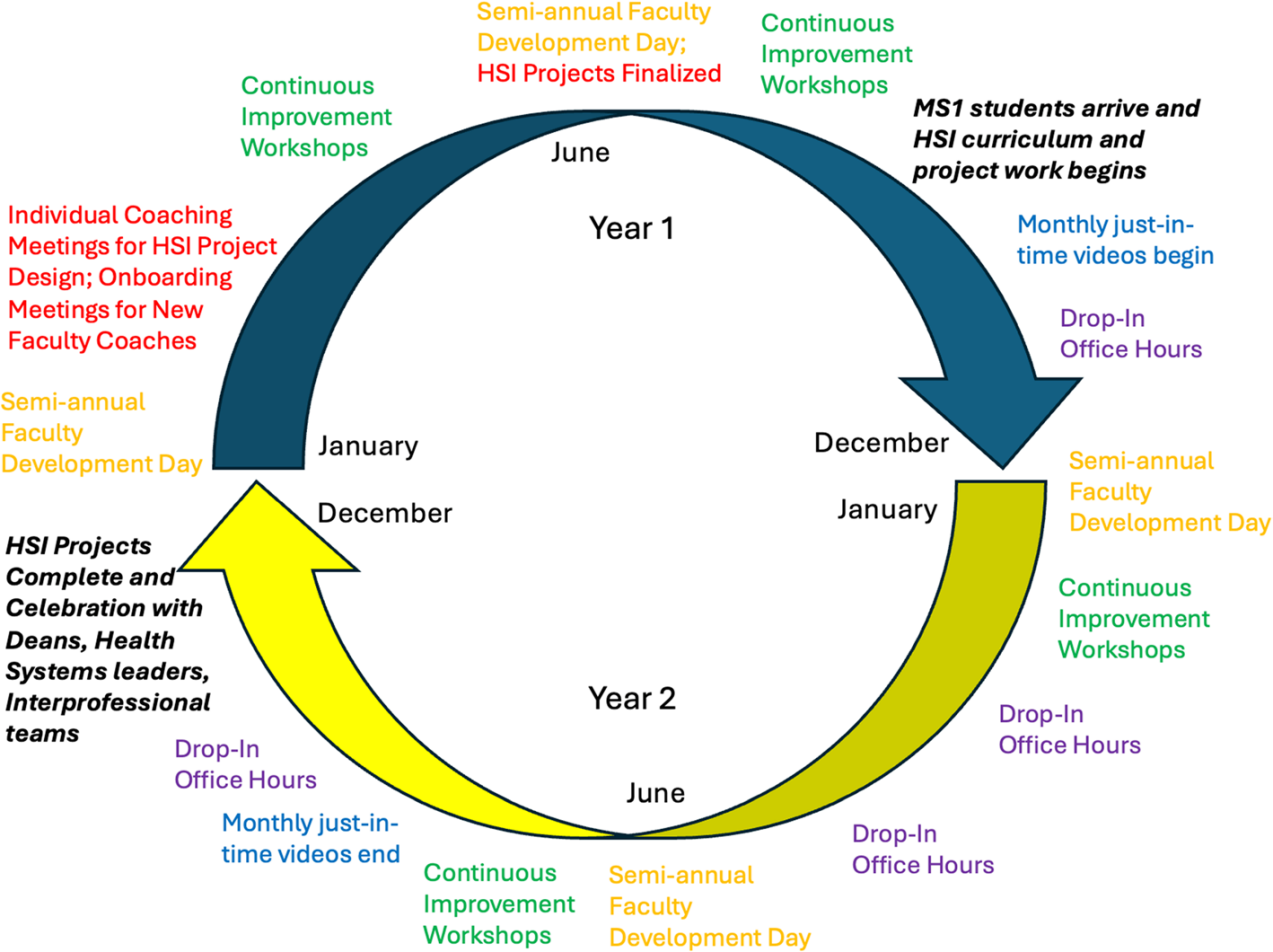
Components and timeline of the HSI faculty development program. The program runs continuously over the course of two years before repeating again for the next cohort of incoming 1 st year medical students. Faculty development leaders run both years of the program simultaneously for two cohorts of faculty coaches. The faculty development program consists of 5 different components: 1) Individual Coaching Meetings to develop longitudinal HSI projects (RED) – In addition to new coach onboarding meetings upon being hired as a faculty coach, all coaches participate in 1–2 meetings with curricular leadership to brainstorm HSI project ideas, design HSI projects in the clinical learning environment that would benefit from the involvement of 5–6 preclinical medical students over the course of 14 months, and finalize project planning through the application of the A3 problem-solving framework. 2) Semi-annual Faculty Development Days (ORANGE) – Content delivered during these sessions include workshops on *Creating an Exceptional HSI Experience, How to Incorporate Health Equity in Your HSI Project, and Demystifying HSI Coaching*. Topics are recommended by and co-designed with curricular faculty development leads and faculty coaches. 3) Continuous Improvement Workshops (GREEN) – These workshops are offered at least four times a year in partnership with our health system’s Continuous Improvement Department. The 4-h *Introduction to A3 Problem Solving* workshop is required for all new faculty coaches, and the remaining 8-h workshops, such as *Leadership and Change Management* are optional. 4) Just-in-time videos to address curricular content (BLUE) – These pre-recorded 5–10-min videos introduce content related to each component of A3 Thinking to faculty coaches on a monthly basis. In addition, 30–50-min faculty development sessions that occur in the weeks prior to relevant HSI small groups workshops are recorded and posted to a central location for all coaches to access asynchronously. 5) Drop-in Office Hours (PURPLE) – Virtual and optional office hours are offered throughout the curricular footprint to provide ongoing support to faculty coaches. Coaches may also reach out to leadership at any point to discuss curricular content and address coach questions and feedback

The aim of the faculty development program is to prepare faculty coaches with the knowledge and skills to teach the HSI curriculum to early undergraduate learners while building and engaging a community of faculty learners. The faculty development program, which was developed by a core team of faculty and staff with a passion for teaching and expertise in systems science and HSI, addresses the needs of faculty in their multi-faceted role, is longitudinal with both standardized and individualized components, and is based upon recommended faculty development best practices of communities of practice, workplace learning, and multi-modal formats with various instructional methods to accommodate different learning styles [16–18]. The HSI faculty development program consists of: 1) individual and group coaching meetings with the curricular team to develop longitudinal HSI projects, 2) semi-annual faculty development days, 3) continuous improvement workshops addressing A3 problem solving and change management, 4) just-in-time videos to address ongoing curricular content, and 5) drop-in office hours and consultation with the curricular team. Many faculty development sessions are recorded, and materials are posted in a centralized location for just-in-time asynchronous access (Fig 1).

### Individual and group coaching meetings

All coaches participate in 3–4 new coach onboarding meetings with curricular leadership, which include an overview of the HSI portion of the curriculum as well as essential components of project selection and design. HSI project design begins seven months prior to the start of each academic year, and requires careful planning and support as faculty coaches are responsible for designing the weekly content of 3 h of HSI curriculum. Appropriate guidance is provided to coaches by five curricular faculty and three staff members with expertise in HSI and medical education to ensure project design is aligned with curricular goals and structure, including interprofessional teaming and partnership, as well as with health system priorities. Support for project design includes guidelines and project development worksheets, an exemplar schedule that maps out HSI curricular work over 14 months, resources to share with clinical team members, and 1–2 meetings with each coach to brainstorm projects, provide constructive feedback, and iteratively design and strengthen the HSI project prior to the students’ arrival. A project design framework that combines a critical thinking and problem-solving framework (A3) with pragmatic operational design of the project experience is utilized in this process [35]. (SUPPLEMENT A) Throughout the academic year, monthly group meetings for the coach cohort supplement these initial individual meetings, offering additional touchpoints for check-in, trouble-shooting, and brief just-in-time faculty development tailored to the progress of student efforts to learn and complete the A3. In addition, coaches participate in ad hoc hour-long faculty meetings where HSI topics are discussed, such as *Student Opportunities for HSI Scholarship* and *Sustainability and Quality Improvement*.

### Semi-annual faculty development days

Twice a year, coaches are required to participate in 5–8-h Coach Days that consistently includes 1.5–4 h of workshop content specific to ongoing development of HSI knowledge and skills along with community building activities that offer opportunity for casual and critical knowledge transfer among coaching peers. Occasionally, Coach Day content includes required HSI content delivered in a plenary session. Examples of workshops delivered at semi-annual faculty development days include *How to Incorporate Health Equity in Your HSI Project, Creating an Exceptional HSI Experience,* and *Demystifying HSI Coaching*.

### Continuous improvement workshops

All new faculty coaches are required to participate in a health system Lean A3 Problem Solving workshop (4 h) offered to all faculty through the Continuous Improvement Department, which is a centralized resource whose mission is to support the strategic deployment of the Lean Management System at one of our health systems. Optional workshops offered include Leadership and Change Management (8 h) and core topics in Lean methodology including Active Daily Management Communication and Alignment (8 h) and Introduction to Continuous Improvement (8 h). Curricular leadership facilitate deliberate communication and planning to ensure workshops align with coach schedules and are offered 3–4 times per year with some sessions tailored specifically for the faculty coach role. Partnering with existing training in one of our health systems allows for continued alignment between the School of Medicine and the health system.

**Fig. 2:**
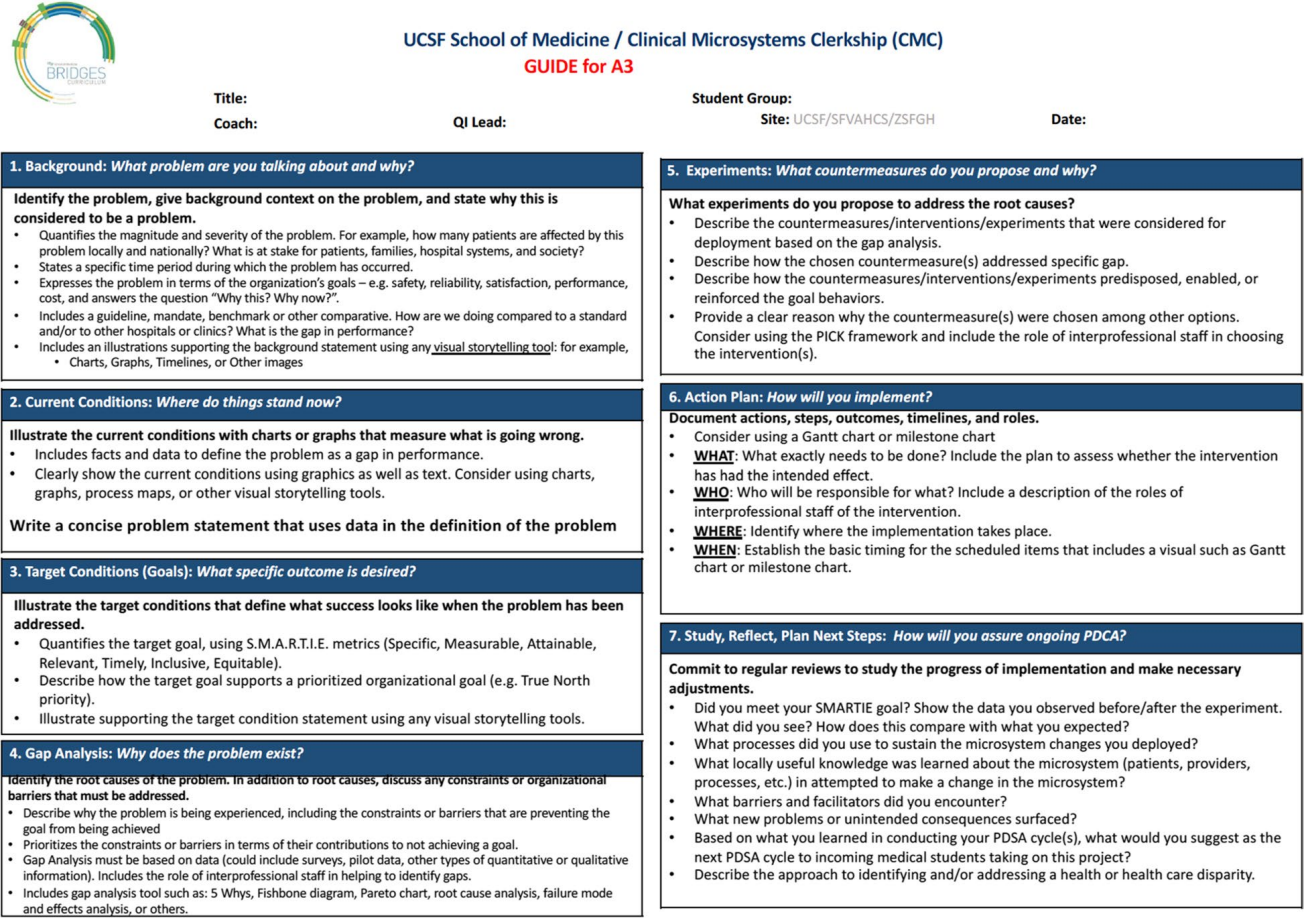
A3 problem-solving rubric: a shared model for HSI and framework for student assessment. The A3 framework is a shared tool that is used for problem-solving at all 3 of our health system sites and the School of Medicine. The A3 is divided into 7 components, and each component has specific criteria that must be met for faculty coach-led student teams to meet expectations at four pre-determined assessment points over a 14-month period: Components 1–3 (Assessment #1), Component 4 (Assessment #2), Component 5–6 (Assessment #3), and Component 7 (Assessment #4)

The A3 Problem Solving workshop is required for new faculty coaches given its central importance in the experiential learning of students in leading systems change in their assigned clinical microsystems, particularly if the faculty coach has not participated in similar previous training. The A3 contains shared language and tools that the three participating health systems and School of Medicine utilize in problem-solving. Additionally, students are assessed on their systems problem-solving skills through the submission of different components of the A3 four times over the course of the HSI curriculum (Fig 2).

### Just-in-time curricular content videos

Coaches participate in asynchronous monthly Just-In-Time “HSI Snacks” that provide ongoing curricular content and key teaching pearls that are relevant to the curriculum via pre-recorded 5–10-min videos. Additional 30–50-min faculty development sessions are offered in the weeks immediately prior to relevant health systems improvement small group content that includes *Introduction to A3 Thinking and Human Centered Design, HSI and Health Care Disparities,* and *Works-in-Progress Session*.

### Office hours and consultations

Drop-in virtual optional office hours are provided 1-h per day over three days at four timepoints throughout the curriculum to offer HSI assessment grading support for coaches. Faculty are never asked to assess their own students. Participating in peer review of other coaches’ and students’ projects provides faculty the opportunity to strengthen their own HSI project skills and learning by providing and receiving peer feedback. As needed, curricular leadership meet 1:1 with faculty coaches to provide guidance on teaching the HSI curriculum, including: managing workplace learning, teaching small group didactics, and integrating HSI assessment for learning into their group’s and students’ experience.

## Program evaluation

We assessed the efficacy of the HSI faculty development program at providing the knowledge and skills for physician educators to achieve competence to deliver a longitudinal HSI medical student curriculum using the Kirkpatrick Model of evaluation (reaction, learning, behavior, impact). We also specifically sought to determine the value, as defined by experience, quality, and retention, of the HSI faculty development program on physician faculty coaches, medical students, and participating health systems. Data was collected prospectively as the HSI faculty development program was implemented. Student assessment outcomes reflect both educator and learner knowledge and skills and uses a project grading rubric aligned with health systems improvement standard processes.

## Results

From 2016 to 2023, 119 physician faculty members in 15 clinical departments across three health systems (academic medical center, safety net hospital, and veterans’ affairs health care system) participated in the UCSF CMC health systems improvement faculty development curriculum. At the time of being hired, 53% of faculty coaches were at the Assistant Professor rank, 30% at the Associate Professor rank, and 17% at the Professor rank. 71% of faculty coaches are in non-procedural specialties and 29% in procedural specialties. There are currently 62 faculty coaches, with 18% self-identified as underrepresented in medicine. During 2020–2023, this program onboarded and trained 49 new faculty physician coaches (average turnover 12% per year). We describe outcomes below based on Kirkpatrick’s model of evaluation.

## Kirkpatrick’s level 1: reaction

Physician coach satisfaction of faculty development workshops is rated an average of 4.7 on a scale of 1 (poor) to 5 (excellent). Qualitative comments about the sessions include: “I honestly think the workshops were so good that I recommend you recycle them…no need to reinvent the wheel each time,” “Faculty development for coaches is exceptional. It is one of the areas where I am happiest as faculty. Nowhere else in my professional life do I get so many opportunities to grow and such great colleagues to learn from,” “Health systems improvement faculty development sessions have been really useful,” and, “[the facilitators for] *Making QI Come Alive* were engaging, warm, and offered some useful resources.” In addition, faculty have universally found the mandatory A3 Thinking workshop to be very useful in preparing for their role as HSI project coaches.

Faculty satisfaction with the physician coach role, of which HSI is one component (in addition to teaching direct patient care skills, providing career guidance, and student coaching), is 4.5 on a scale of 1–5.

Student rating of coaches is 4.8 on a scale of 0–5.

## Kirkpatrick’s level 2: learning

From the time of starting in the role, mostly with little to no knowledge of HSI skills, to the time faculty coaches work with students, coaches demonstrate and apply their knowledge through HSI project design and implementation. Each physician coach submits a project design workbook for two possible HSI projects prior to matriculation of each new entering class of MS1s. The project design process includes requirements for a successful project and requires faculty coaches to draft an A3 for the health care problem students will be working on over the course of 14 months and begin planning for weekly 3-h sessions.

Through coaching, iterative project development, and individual follow-up, *all* faculty coaches develop sufficient competence to teach HSI to MS1s and MS2s, which is demonstrated through successful project implementation that includes at least one full Plan-Do-Study-Act cycle and applying principles of change management and stakeholder engagement.

Coaches also demonstrate their learning by grading other students’ A3 submissions using a standard grading rubric (Fig 2). Students submit one A3 per project, and 30–40 A3s are submitted at four assessment points over the course of the curriculum for each class of students. Coach concordance with curricular leader grading is high (> 90%), as program leadership verify the accuracy of each assessment through a secondary evaluation. All students met expectations at course conclusion.

## Kirkpatrick’s level 3: behavior

Faculty coaches demonstrate behavior change in two ways. First, faculty engage in the project planning process to design and create a HSI project and plan the activities using a curricular instructional framework. This planning process that is unique to each faculty coach enables them to teach a total of 35 small group sessions that are 3 h each over 14 months, for a total of 105 HSI instructional hours per faculty, which is significantly greater than any prior faculty’s HSI teaching experience prior to engaging in the HSI faculty development program. Second, some faculty coaches choose to enroll in a separate, intensive weeklong, 8 h per day workshop in our health system that covers principles of continuous improvement, including the Lean management system, Lean tools, and leadership development. A total of 27 faculty coaches have successfully completed this training program.

## Kirkpatrick’s level 4: impact

### Impact on physicians

Measurable impact on physician faculty coaches includes job retention, promotion, and recognition. Many faculty coaches describe this role as a key reason for staying at the academic institution. From 2020–24, 22% of faculty coaches were promoted to leadership positions in quality improvement or clinical operations (*n* = 26/119), with their experience as coaches providing experiential HSI learning for medical students being a significant contributor to their being chosen for these leadership positions. In addition, 43% of coaches (*n* = 13/30) who have chosen to leave the program have been promoted internally within the institution to positions in education or clinical leadership. There are 29 current and former coaches (24%) who have been competitively selected as members of the UCSF Academy of Medical Educators, a recognition of their outstanding skills as academic educators that for many meets criteria for accelerated promotion. Although job retention, promotion, and recognition cannot be solely tied to or attributed to a faculty coach participating in the HSI faculty development program, coaches share that this program is essential and critical to develop and improve their HSI competency as a faculty coach. This is a unique and distinguishing skillset that is of value to medical education and health systems, and is recognized as a significant achievement in medical education at our institution.

### Impact on students

Student assessment outcomes for the initial submission of the four A3 assessments were: 57–89% met expectations for Components 1–3 (Background, Current Condition and Problem Statement, Target Condition), 70–100% met expectations for Component 4 (Gap Analysis), 67–95% met expectations for Components 5–6 (Experiments, Action Plan), and 90–95% met expectations for Component 7 (Study/Act). All students met expectations across all A3 components; for some, based on required re-submission after not meeting expectations initially. 100% of student teams submit a poster at the HSI Symposium at the end of the MS2 year, and up to 15% of student teams participate in other scholarly activity, including submission of peer-reviewed manuscripts or abstracts to national or international meetings. All students’ residency application Medical Student Performance Evaluation letters include a description of their HSI projects.

Students are surveyed during their final year of medical school regarding the impact of the HSI curriculum on their continued learning and professional identity formation as a physician. 97% responded that they believe physician clinical skills and health systems knowledge are both important to good patient experience and clinical outcomes. 71% of students agreed and strongly agreed that they envision that HSI will be a part of their future work as a physician and 51% of students agreed and strongly agreed that the HSI curriculum prepared them for success in their clerkships and career launch clinical rotations. For instance, one student wrote, “learning the approach to quality improvement helped me constantly think through how I can provide better care to my patients during clerkships. I am now interested in pursuing a career that involves quality improvement at the clinical level.”

### Impact on health systems

Since program inception, physician coaches have guided 1,281 students to spend 134,400 h on 242 health system improvement projects in multiple departments and levels of care within three health systems. Sample projects are listed in Table 1. All projects are aligned with our health systems’ priority areas, which are quality [*n* = 160, 66%], health equity [*n* = 46, 19%], safety [*n* = 21, 9%], patient experience [*n* = 18, 7%], financial strength [*n* = 3, 1%], and strategic growth [*n* = 3, 1%].

**Table 1:** Examples of health systems improvement projects categorized in alignment with institutional priority areas

Institutional priority area	Project name	Clinical setting	Project impact and results
Quality	*Increasing Access to Routine Immunizations at the Pediatric Acute Care Clinic*	Pediatric Urgent Care Clinic	Decreased the rate of routine immunization care gaps from 28 to 20% within 1 year
	*Improving Primary Care Referrals for Fentanyl Test Kits for Veterans*	Veterans Affairs Primary Care Clinic	Improved utilization of the Fentanyl Test Kit Distribution Consult service by 100% using a provider and patient-focused education campaign
	*Increasing Advanced Care Planning in Post Lung Transplant Patients*	Sub-Specialty Clinic	Patient education that empowers post-lung transplants patients to complete their advanced care planning improved rates from 17.7% to 40%
Health care equity	*Addressing Food Insecurity in a Family Medicine Clinic*	Primary Care Practice	Discovered that 12.4% of clinic patients screened positive for food insecurity. Implemented a food voucher program and provider education campaign to increase food pharmacy attendance from an average of 10.3 to 19.8 weekly participants
	*Improving Access to Care Through MyChart Enrollment in the Pediatric Asthma/Allergy Clinic*	Pediatric Asthma and Allergy Clinic	Improved average monthly MyChart enrollment for all patients by over 20%, with specific outreach to Spanish speaking patients, by providing in-person enrollment support through an existing volunteer program
Safety	*Clamp Down on Central Line Associated Bloodstream Infections*	Adult Intensive Care Unit	Decreased CLABSI rate from 0.85 to 0.5 by implementing a CLABSI bundle checklist and dressing educational campaign
Patient experience	*Improving Care Delivery and Experience for Transgender and Nonbinary Patients in the Psychiatric Inpatient Unit*	Inpatient Psychiatry Unit	Designed and implemented a training video to improve staff knowledge of how to implement gender-affirming care practices
	*Improving the Hospitalized Patient Experience for Individuals with Limited English Proficiency (LEP)*	Adult Inpatient	Partnered with design experts to interview patients with LEP to inform next step of piloting a hospital orientation video for patients with LEP. Patient experience scores improved from 84 to 92%
Financial strength	*Environmental Sustainability and Waste Reduction in the Operating Room*	Perioperative Services	Operating room waste greatly contributes to climate change. Eliminated $24,170 of wasted supplies by updating supply lists for thyroid operations
Strategic growth	*Emergency Department Jade*	Adult Emergency Department	Optimized workflow to care for moderate acuity patients in the Emergency Department and decreased average ED arrival to provider evaluation time from 115 to 81 min

### Discussion

This study demonstrates that a longitudinal, multi-faceted faculty development program successfully trains physician faculty to effectively engage early medical students in systems improvement efforts in the clinical environment amongst interprofessional teams. Physician and learner satisfaction levels were high. Sufficient knowledge, skills, competence were achieved, and desired behavior change along with impact on the health system was demonstrated within the clinical learning environment. Notably, the outcomes reported here that map to Kirkpatrick level four – impact on educators, students, and health systems – are infrequently reported in medical education and faculty development studies [16].

We demonstrate that a train as you teach model is feasible and effective, even for a topic that is relatively unfamiliar to many physicians. This program was designed to address the gap that most practicing physicians today have had little to no health systems science training [36]. Similarly, most of our faculty coaches began their roles with little systems improvement experience. Hence, our faculty development program was designed to enable coaches to co-learn the material with peers, and just ahead of their learners. This model is pragmatic given the lack of available and experienced HSI faculty in most medical schools and provides a generalizable model for institutions to increase the competence of existing faculty in teaching health systems science and HSI. This aligns with calls for “new” educators in health systems science in our clinical environments by developing the educational skills of existing clinician educators to include HSS-related concepts and skills [23]. In this way, an education community of practice emerged.

Designing a faculty development program that allows for faculty to co-learn material just ahead of their learners is also beneficial in allowing curricular leaders to continuously and iteratively improve the curriculum based on learner and faculty feedback. This ability to pivot relatively quickly in training faculty can also be beneficial when there may be shifting approaches and priorities to improvement work within health systems. The training model of learning new material before teaching it is also one that is familiar to faculty as it is what the medical profession’s training model is based upon: see one, do one, teach one. Training faculty to develop competencies and skills in teaching and coaching HSI is no different.

In addition, there are several key elements of our HSI faculty development program that are based on best-practices in competency-based faculty development and are success factors for sustaining this program over nearly a decade to date. First, the program is deliberately longitudinal and spaced out over time with greater time spent on foundational knowledge and competence in the first few months after new faculty coach onboarding [17, 18]. Content is delivered through multiple modalities including in-person workshops and meetings, individual coaching meetings with faculty development leads, drop-in office hours, and online video content. Faculty development is frequently conducted a few weeks prior to teaching and coaching curricular content to learners, and faculty coaches have access to the community of approximately 60 coaches who are similarly engaged and motivated to be medical educators in HSI. Being able to engage and learn with like-minded peers in communities of practice is an extraordinarily motivating factor that predisposes faculty to want to participate further in their own development in HSI, which is a key facilitator informed by adult learning theory [19, 37].

A second critical element is the strategic alignment between our faculty development program (based in the education mission) with our health system partners (core to the clinical mission) [38, 39]. This begins with a shared systems improvement framework between our health system partners and the School of Medicine. All entities adopted the Lean management system over a decade ago and all utilize A3 thinking as the methodology for approaching problem-solving in the spirit of continuous improvement. Without this alignment and shared language for engaging medical students and interprofessional teams in HSI, the approach to curricular content and faculty development would have been much more challenging.

The next strategy is regular meetings with health system senior leadership to engage in bi-directional conversations about the HSI medical student curriculum and health systems’ priorities [39]. These bridging conversations between leaders in medical education and our health systems has resulted in continued interest, appreciation, and celebration of the work medical students are doing to improve clinical care. We host an annual symposium celebrating the culmination of the 14-month HSI curriculum where all project teams present posters of their work. Health systems and microsystem leaders attend to learn about the impact of faculty coach and student led improvement work on patient care.

Furthermore, elevating the importance and impact of pre-clinical medical student and faculty coach work in improving clinical care delivery allows health systems leaders to see the tangible and concrete value of supporting medical education [20]. By guiding students through a longitudinal HSI curriculum and systems improvement project, faculty coaches are able to demonstrate their knowledge and skills in systems thinking, change management, interprofessional teaming, and health systems leadership, which are core competencies that leaders in academic health centers have identified as being required for faculty to transform health care [40]. This can have a big upside at academic medical centers where there are existing tensions in funding the clinical enterprise, education, and research missions [41, 42].

Developing faculty in HSI and health systems science skills can lead to increased professional fulfillment, satisfaction, and promotion, which can be effective in addressing the crisis of medical professional burnout by creating increased meaning in work [43]. The leaders in both medical education and health systems strongly believed in and continue to see the benefits of empowering faculty physicians to teach and coach early medical learners in health systems redesign at the level of our varied clinical microsystems. This level of continued commitment is essential to begin and sustain a similar HSI faculty development program.

This report has limitations. Our exploratory study was conducted at one institution with three affiliated health systems that all include a mission for education. This program was developed alongside a broader curricular redesign effort that included a realigned education organization and educator funding structure. Since the faculty coach role encompasses multiple aspects, including teaching and mentoring, not all satisfaction outcomes can be considered directly causal.

Despite the intensive efforts and successes of the HSI faculty development program, faculty coaches provide anecdotal feedback that teaching HSI requires significant time, energy, and investment in mastering the knowledge and skills needed, and many continue to find the HSI portion of their teaching role to be hard. Guiding students in systems improvement work within dynamic clinical microsystems, where the change management skills needed to redesign clinical operations and workflows towards an improvement goal are demanding, remains daunting in spite of this well-received faculty development program. This observation speaks to the realities and challenges of innovation within our health systems in general.

The current study includes perspectives from educators and learners but lacks the input of health system stakeholders, which could be a next step. Other potential avenues of investigation include following the longer-term career path and trajectory of prior and current faculty coaches and determining their self-reported impact of the HSI faculty development program on their career and job satisfaction. Lastly, the ability for a faculty development program to have similar successful outcomes without comparable funded support for faculty remains unknown.

### Conclusions

A longitudinal and multi-faceted faculty development program is feasible, pragmatic, and successful in developing faculty physicians to have the knowledge, skills, and necessary tools to effectively coach and lead early medical students in systems change in the clinical learning environment. This faculty development program addresses a need of medical school faculty, and is focused on physician faculty gaining competence in health systems science such that all faculty who participate are able to effectively engage medical students in health systems science and HSI. Such a program can offer alignment between the education and clinical missions in academic health care systems.

## Supplementary Information

Supplementary Material 1.

## Data Availability

The data in this study are available upon request.
