# Supplementary material for: Bridging early medical education and health systems improvement: a multi-faceted faculty development program to enhance engagement and impact
Source: BMC Med Educ. 2025 Jul 25;25:1112. doi: 10.1186/s12909-025-07579-9 (PMC12291519; doi:10.1186/s12909-025-07579-9)
Supplement: Supplementary file 1 — Supplementary Material 1. [file 12909_2025_7579_MOESM1_ESM.docx]

**
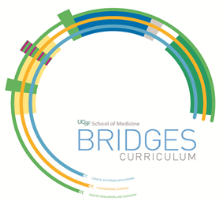
 Clinical Microsystems Clerkship (CMC)**

**Health Systems Improvement (HSI)**

**Project Planning Packet**

**Part One - Project Development: Required Criteria and Submission Process**

**Project Overview:**

The 21st century physician must be ready to participate effectively in improving quality, patient experience, safety, healthcare value, and equity to achieve the aims of high-quality healthcare. This requires a deep understanding of healthcare systems that is only achieved through adopting authentic roles in real-world system improvement efforts. To advance early medical students’ knowledge and skills in health systems, the University of California, San Francisco School of Medicine (UCSF) Clinical Microsystems Clerkship pairs small groups of first-year medical students with a faculty clinician “coach” to conduct project-based systems improvement work in a clinical setting, utilizing a systematic and interprofessional team-oriented approach to improve the quality and value of care for patients and populations.

**Project Criteria:**

Based on recent years of student health systems improvement experience, including past coach and student feedback, successful projects have met ALL of the following criteria:

**Required:**

- Clear link to organizational/institutional goals including equity and healthcare disparities
- Data stream available to implement/inform project
- Interaction with Interprofessional team members
- Feasibility to implement over approximately 14 months. If the initial project has significant challenges OR if the project timeline may not align with the CMC 14 month timeline, it is acceptable to pivot to different project as long as student teams experience *at least one* full Plan-Do-Study-Act (PDSA) cycle.
- Early student interaction with patients
- Authentic and adapting student role/responsibilities and learning (not a static student role) over time
- Space/resources available for students to work on their projects (computers, etc)
- Designated longitudinal QI project lead – This individual is responsible for overall project oversight. This can be the coach or a different designated QI project lead. If the QI project lead is not the coach, please consult, follow, and share [this document](https://courses.ucsf.edu/pluginfile.php/367721/mod_page/content/120/2022%20HSI%20Project%20Lead%20Description.docx?time=1674776949461) that outlines the roles and responsibilities of the QI Project Lead.
  - Recommended: Project lead has QI experience and either agrees to be the QI lead or can identify their QI resource
- 1 project per coach: highly recommended for all coaches and required for new coaches
- Lean A3 Training (February 5 and April 16 @Mission Bay): highly recommended for all coaches and required for new coaches

**Coach Project Proposal Development and Submission Process:**

- Now: Review the detailed timeline [here](https://courses.ucsf.edu/mod/page/view.php?id=224146)
- **January 2024: First of two 1:1 meetings with CMC Leadership – Brainstorm and project selection discussion.** Returning coaches are invited to reflect on the following questions in advance of this meeting:
  1. What lessons have you learned from previous projects?
  2. What do you want to apply this next time around?
- **April 19, 2024: Submit Phase 1 of worksheets** (available below) – Formal project proposal
- **April-May 2024: Second of Two 1:1 meetings with CMC Leadership –** Discussion of proposed project(s) submitted in Phase 1 of worksheets; Project confirmed
- **May-June 2024 (Optional, depending on results of April meeting):** 1:1 meeting with CMC Leadership - Project confirmed
- **June 7, 2024: Submit Phase 2 of worksheets** (available below)

**WORKSHEETS: Health Systems Improvement (HSI) Project Proposal**

**& Accompanying Microsystem**

**Questions? Please reach out to the CMC HSI Leadership Team**.

First Meeting: January/February 2024 – Brainstorm and Discussion Notes

[*Please review your institution’s* True North/Organizational Goals](https://courses.ucsf.edu/mod/page/view.php?id=224609) *and the project criteria outlined on page one of this document. While considering these items, please use the space below to share your current thoughts about projects you are considering, thinking about, in conversations about, etc. We also would like to understand your successes and challenges in HSI project coaching, and if you are new to coaching, we are here to help guide and support you in the HSI project design process. We want to get a sense of what your current thoughts are – they may be well-developed or they may be surrounded by question marks.*

[Site Specialist will take meeting minutes and send to coach after first 1:1 meeting in Jan/Feb]

Phase One: Due April 19, 2024 (Required)

*Please respond to all prompts below, providing details about two proposed projects and their accompanying microsystems.* ***Please submit two project proposals****: ideally, you will only fully develop one project for your entire student team. However, researching a minimum of two projects have proven beneficial to both (1) identify the best possible project for your students and (2) develop a back-up project should your chosen project fall through.*

PROJECT PROPOSAL ONE

Respond to all questions below.

**HSI PROJECT PROPOSAL #1: Project Details (required)**

1. Project Title:
2. Number of Students that should be assigned to this project, min/max (ideal):
3. In sections A-G below, describe what you would like your students to improve in the microsystem.
   1. Brief Background/Literature: Why this problem? Why improve the problem now?
   2. Current State: What do you know? Are there existing data to justify the need for improvement? If there are not existing data, describe/draw a process flow map of the inter-professional team workflow that illustrates the need for improvement.
   3. Draft Target SMARTIE Goal:

- Is a healthcare disparity present for the population you are studying? How will you assess for the presence of a healthcare disparity in the population you are studying?
  1. Institutional Goal this QI Initiative relates to:
  2. Possible barriers and gaps that are preventing you from achieving your goal:
  3. Proposed Experiment #1:
  4. Proposed Experiment #2:

1. Briefly describe the data available. If there are not existing data, how can existing data be modified to better suit the HSI project’s needs?
2. In sections A-C below, describe how will your students know if there has been a change as a result of their project (i.e. what specific things will you measure).
   1. Was the experiment conducted (i.e. in-process measure)?
   2. Was any part of the target achieved (i.e. primary outcome measure)?
   3. Were there unintended consequence of improvement work (i.e. improving antibiotic administration time for patients presenting with sepsis may result in increased antibiotic resistance patterns and/or C. difficile infections)?
3. Name and QI role of QI Lead(s) who will work with students (list individual titles):
4. Name Interprofessional Team member(s) that will work with students:
5. How will the additional staff and faculty engage with the students? (i.e. orientation, supervision, coaching or mentoring, participation in QI efforts)
6. How will you inform these individuals about the CMC, student’s roles, and learning objectives?
7. What will be the role of the student in quality improvement (QI) or systems improvement (SI) over 14 months? List specific learning objectives and learning activities for each improvement effort.
8. How and when will students interact with patients?

Additional Notes:

**HSI PROJECT PROPOSAL #1: Microsystem details (required)**

1. What is the microsystem students will be working in (describe specific clinic/department)?
2. Number of students microsystem can accommodate:
3. Specific reporting address for students:
4. Hospital/Site:
5. Address:
6. Building Name/Number:
7. Floor Number:
8. Room Number:
9. Zip Code:
10. What microsystem-specific knowledge and skills do students need to learn on their first day(s)?
11. On-boarding requirements (special badge access? EHR access? Etc.)
12. EHR access: at UCSF Health and at ZSFG, we can request higher-level access (MS3/MS4 access – editing ability beyond the default read only access for MS1s) for students to the relevant EHR. This requires the students to complete extensive training and the coach to ensure students are trained with how to interact with the EHR in alignment with policy and legal regulations. If your project requires this access, please request that here:
13. Rules of the workplace
14. Space for students
15. Patient population overview
16. Learning objectives for each QI or systems improvement effort.
17. Does a systems/quality improvement office exist at your microsystem? What is the SI or QI infrastructure external to the microsystem that students can connect with? How?

Other notes (please include any factors unique to your microsystem, for example student gender/language requirement):

PROJECT PROPOSAL TWO

Respond to all questions below.

**HSI PROJECT PROPOSAL #2: Project Details (required)**

1. Project Title:
2. Number of Students that should be assigned to this project, min/max (ideal):
3. In sections A-G below, describe what you would like your students to improve in the microsystem.
   1. Brief Background/Literature: Why this problem? Why improve the problem now?
   2. Current State: What do you know? Are there existing data to justify the need for improvement? If there are not existing data, describe/draw a process flow map of the inter-professional team workflow that illustrates the need for improvement.
   3. Draft Target SMARTIE Goal:

- Is a healthcare disparity present for the population you are studying? How will you assess for the presence of a healthcare disparity in the population you are studying?
  1. Institutional Goal this QI Initiative relates to:
  2. Possible barriers and gaps that are preventing you from achieving your goal:
  3. Proposed Experiment #1:
  4. Proposed Experiment #2:

1. Briefly describe the data available. If there are not existing data, how can existing data be modified to better suit the HSI project’s needs?
2. In sections A-C below, describe how will your students know if there has been a change as a result of their project (i.e. what specific things will you measure).
   1. Was the experiment conducted (i.e. in-process measure)?
   2. Was any part of the target achieved (i.e. primary outcome measure)?
   3. Were there unintended consequence of improvement work (i.e. improving antibiotic administration time for patients presenting with sepsis may result in increased antibiotic resistance patterns and/or C. difficile infections)?
3. Name and QI role of QI Lead(s) who will work with students (list individual titles):
4. Name Interprofessional Team member(s) that will work with students:
5. How will the additional staff and faculty engage with the students? (i.e. orientation, supervision, coaching or mentoring, participation in QI efforts)
6. How will you inform these individuals about the CMC, student’s roles, and learning objectives?
7. What will be the role of the student in quality improvement (QI) or systems improvement (SI) over 14 months? List specific learning objectives and learning activities for each improvement effort.
8. How and when will students interact with patients?

Additional Notes:

**HSI PROJECT PROPOSAL #2: Microsystem details (required)**

1. What is the microsystem students will be working in (describe specific clinic/department)?
2. Number of students microsystem can accommodate:
3. Specific reporting address for students:
4. Hospital/Site:
5. Address:
6. Building Name/Number:
7. Floor Number:
8. Room Number:
9. Zip Code:
10. What microsystem-specific knowledge and skills do students need to learn on their first day(s)?
11. On-boarding requirements (special badge access? EHR access? Etc.)
12. EHR access: at UCSF Health and at ZSFG, we can request higher-level access (MS3/MS4 access – editing ability beyond the default read only access for MS1s) for students to the relevant EHR. This requires the students to complete extensive training and the coach to ensure students are trained with how to interact with the EHR in alignment with policy and legal regulations. If your project requires this access, please request that here:
13. Rules of the workplace
14. Space for students
15. Patient population overview
16. Learning objectives for each QI or systems improvement effort.
17. Does a systems/quality improvement office exist at your microsystem? What is the SI or QI infrastructure external to the microsystem that students can connect with? How?

Other notes (please include any factors unique to your microsystem, for example student gender/language requirement):

Phase Two: Due June 7, 2024

All coaches are encouraged to complete this section independently in preparation for student arrival and new coaches are required to do so. You do not need to submit your responses to these questions to the CMC Leadership team, yet you are welcome to do so if helpful.

**Project Implementation Planning:**

All coaches are expected to familiarize themselves with the CMC HSI **Exemplar Project Schedule** for New HSI Projects, which is updated periodically throughout the academic year. Please do the following prior to June 7, 2024 and continue to return to this activity throughout the summer and the upcoming academic year:

- Access the exemplar schedule [here](https://courses.ucsf.edu/course/view.php?id=3248)
- **Map out the first 4-8 weeks of microsystem time for your students and project(s). This includes the weeks of:**
  - **Sep 16-20: Week 1/Immersion Week. There will be several REQUIRED activities to complete during Immersion Week and there may be no more than 1-1.5 hours of time to conduct project-specific work.**
  - **Sep 23-27: Week 2**
  - **Sept 30-Oct 4: Week 3**
  - **Oct 7-11: Week 4**
  - **Oct 14-18: Week 5**
  - **Oct 21-25: Week 6**
  - **Oct 28-Nov 1: Week 7**

**Design of the Student Workplace Experience:**

Recommendation to brainstorm/reflect on these questions and jot down some ideas. New coaches might discuss these with your Site Director and/or a coaching peer to get ideas for how to design the student workplace experience. There is no formal requirement to submit written responses to these thought prompts.

1. What will be done to provide a welcoming and inviting environment for students?
2. Who should they meet virtually/in-person and how often?
   1. If you are not the QI Project Lead, how often will you meet with the QI lead to discuss student progress in the HSI project (ideally every 1-2 weeks)? How often will the students meet with the QI lead (ideally weekly)?
3. How will the students be integrated into the microsystem/s to contribute in meaningful ways?
4. How independently/proactively should the students act?
5. What knowledge and skills are students expected to develop while in the microsystem and who will guide this?
6. What other learners (peers, residents, other health professionals) will be in the microsystem and how will interactions be encouraged?
7. How will the students’ time in the microsystem generally be structured?
8. What clinical and systems-oriented activities will be incorporated?
9. Which important microsystem/staff meetings should the students attend? Do they fit with the students’ schedule?

**Questions? Please reach out to the CMC HSI Leadership Team.**
